# Corneal Wound Healing in the Presence of Antifibrotic Antibody Targeting Collagen Fibrillogenesis: A Pilot Study

**DOI:** 10.3390/ijms241713438

**Published:** 2023-08-30

**Authors:** Zeba A. Syed, Tatyana Milman, Jolanta Fertala, Andrzej Steplewski, Andrzej Fertala

**Affiliations:** 1 Wills Eye Hospital, Philadelphia, PA 19107, USA; zsyed@willseye.org (Z.A.S.); tmilman@willseye.org (T.M.); 2 Department of Orthopaedic Surgery, Sidney Kimmel Medical College, Thomas Jefferson University, Curtis Building, Room 501, 1015 Walnut Street, Philadelphia, PA 19107, USA; jolanta.fertala@jefferson.edu (J.F.); andrzej.steplewski@jefferson.edu (A.S.)

**Keywords:** cornea, scarring, collagen, proteoglycans, therapeutic antibody, antifibrotic, wound healing, eye disorders

## Abstract

Highly organized collagen fibrils interlacing with proteoglycans form the crucial architecture of the cornea and facilitate its transparency. Corneal scarring from accidental injury, surgery, or infection alters this highly organized tissue, causing severe consequences, including blindness. There are no pharmacological or surgical methods to effectively and safely treat excessive corneal scarring. Thus, we tested the anticorneal scarring utility of a rationally designed anticollagen antibody (ACA) whose antifibrotic effects have already been demonstrated in nonocular models. Utilizing a rabbit model with an incisional corneal wound, we analyzed ACA’s effects on forming collagen and proteoglycan-rich extracellular matrices in scar neotissue. We used microscopic and spectroscopic techniques to quantify these components and measure crucial parameters characterizing the structure and organization of collagen fibrils. Moreover, we analyzed the spatial distribution of collagen and proteoglycans in normal and healing corneas. Our study demonstrated significant changes in the quality and quantity of the analyzed molecules synthesized in scar neotissue. It showed that these changes extend beyond incision margins. It also showed ACA’s positive impact on some crucial parameters defining proper cornea structure. This pilot study provides a stepping stone for future tests of therapeutic approaches that target corneal extracellular scar matrix assembly.

## 1. Introduction

Collagen-I-rich fibrils are fundamental elements that form the three-dimensional architecture of connective tissues. Other collagen types, including collagen III and V, coassemble with collagen I molecules to form heterotypic fibrillar structures. Although these collagen types constitute a relatively small portion of the fibrils, they maintain proper fibril diameter and spacing [1,2,3,4,5]. In addition to the collagenous component, proteoglycans (PGs) play a crucial role by organizing collagen fibrils and facilitating their hydration [6].

Although various connective tissues may share a similar molecular composition, they perform different mechanical functions. For instance, tendons transmit forces along their longitudinal axes from the muscle to the skeletal system, while the skin stretches in all directions. The dispersed collagen II fibrils associated with PGs resist compressive forces imposed on the cartilage in the articular joints. Further, different tissues may have remarkably similar collagen-fibril-based compositions, but their diverse functions are possible due to the tissue-specific organization of their fibrillar architecture. For instance, the parallel alignment of the fibrils in the tendon allows its unidirectional-force-transmitting function. In contrast, the random organization of collagen fibrils in the cartilage facilitates its ability to resist compression forces [1].

One of the tissues with highly organized collagen fibrils is the corneal stroma, in which the fibrils have uniform diameter and spacing [2,3,4,5,6]. The parallel fibrils are arranged in layers of lamellae positioned orthogonally to each other. This organization is preserved through the curved cornea, facilitating its transparency. Corneal PGs, most notably keratan sulfate, maintain the proper organization of collagen fibrils and facilitate their hydration [7]. In contrast, the adjacent sclera is opaque despite a similar composition, mainly due to varying diameters of the collagen fibrils and their random spatial organization [8].

As with other connective tissues, injuries to the cornea cause a wound-healing response. During this process, inflammatory factors activate the corneal keratocytes, prompting them to produce scar tissue mainly consisting of collagen-I-rich fibrils [9,10]. Although scar formation is a natural response that maintains the structural integrity of injured corneas, corneal scar tissue may cause severe problems depending on its extent and location, including irregular astigmatism, opacification, and vision impairment [10]. These problems are because scar tissue lacks the crucial organization of the native cornea tissue’s fibrillar architecture, leading to corneal opacity [11].

Therefore, preventing excessive scar formation is a rational approach to reducing the risk of unwanted consequences of corneal injuries. Although numerous methods have been tested to achieve this goal, no adequate antiscarring agents have been developed thus far [12,13].

Because of their high target specificity and safety, monoclonal antibodies are particularly attractive therapeutic biologics [14]. They treat various diseases, including cancer, immunologic disorders, Alzheimer’s disease, certain ocular conditions, and many others [15,16,17,18].

Antibody-based therapies have also been tested for their potential to limit fibrotic scarring. For example, antibodies targeting TGFβ1 and connective tissue growth factor (CTGF) have been investigated to reduce fibrotic scarring in various tissues and organs. Similarly, therapeutic antibodies have been used to target αv integrins, which mediate profibrotic signaling, and lysyl oxidases, which catalyze the cross-linking of collagen fibrils to stabilize scars [19]. However, a recent review by Fertala et al. indicated that targeting these profibrotic factors in vivo did not significantly reduce excessive scar formation [19].

Our research group identified collagen fibrillogenesis as a promising target for limiting excessive scarring [20]. As a result, we developed a monoclonal antibody called anticollagen antibody (ACA) that effectively blocks this process [21]. ACA explicitly targets critical collagen telopeptide domains that drive the assembly of fibrils, which are the main components of scar tissue [20].

Here, we employed ACA, which reduces collagen fibril formation and scar tissue accumulation in many wound healing models, in a rabbit model of corneal injury to study its impact on the composition and organization of two crucial elements of the corneal matrix, namely, collagen and PGs [19,20,21,22,23]. We aimed to delineate the ACA-dependent effects of reducing collagen fibrillogenesis that occurs during the healing of corneal incisional wounds in a rabbit model. Microscopic and spectroscopic methods applied in our study shed new light on changes in these components.

## 2. Results

### 2.1. Animal Model

A total of 10 New Zealand White rabbits (5 male and 5 female) between 8 and 12 months old were employed for this study. The rabbits’ eyes were divided into the following groups: (i) injured and treated with ACA (ACA group, number of eyes *n* = 6), (ii) injured and treated with PBS only (Ctr group, number of eyes *n* = 4), and (iii) uninjured and nontreated (Un group, number of eyes *n* = 10).

**Figure 1:**
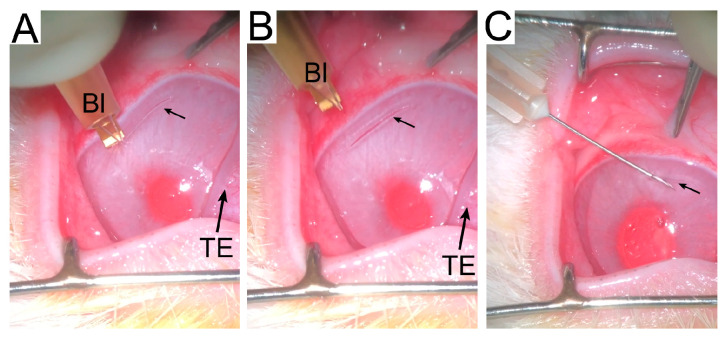
Procedures to create an incisional wound ((**A**–**C**) small arrows) and inject ACA (**C**). The blade (Bl) used to create the incisional wounds and the third eyelid (TE) are indicated.

All animals underwent a unilateral procedure to create an incisional wound (Figure 1). We observed no adverse reactions to the injury or the treatment with ACA. All eyes were utilized for in-life measurements and other assays of the dissected corneas. We measured crucial parameters to characterize scar neotissue formed in the wounded sites and determine the impact of ACA on the scar formation process. The study focused on collagen and PGs, which form the bulk of the corneal scar tissue.

### 2.2. In-Life Assessment of Corneal Scars

Analysis of crucial parameters characterizing the injured corneas was performed in a blinded fashion by an experienced ophthalmologist (Table 1). Mann–Whitney U test was conducted to determine whether there was a difference in the score distributions between the ACA-treated and Ctr groups for depth, opacity, edema, and vascularization of the injury sites measured eight weeks after injury.

**Figure 2:**
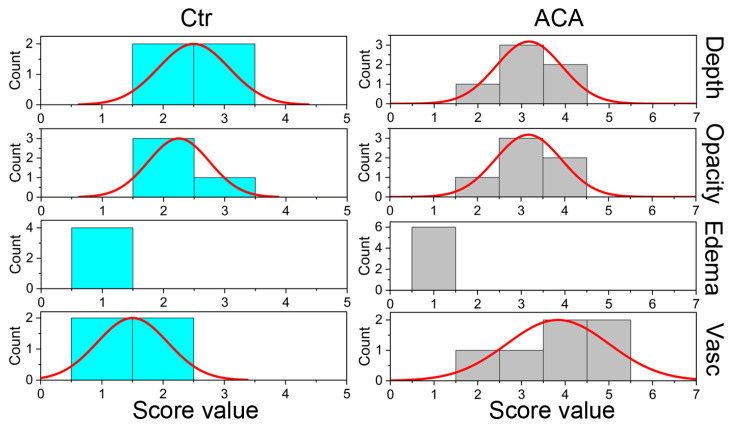
Histograms depicting the distribution of the scores for depth, opacity, edema, and vascularization (Vasc) measured in life eight weeks after creating corneal injuries. Data for the control (Ctr) and ACA-treated (ACA) groups are presented.

As indicated in Figure 2 and Table 2, the distributions of depth, opacity, and edema scores for the ACA and Ctr groups were similar. Regardless of insignificant differences, however, we observed that, except for edema, the scores for the Ctr group trended lower than the ACA group. In contrast, the distribution of the vascularization scores for the ACA (mean rank = 7.33) and Ctr (mean rank = 2.75) groups were significantly different (Figure 2).

**Figure 3:**
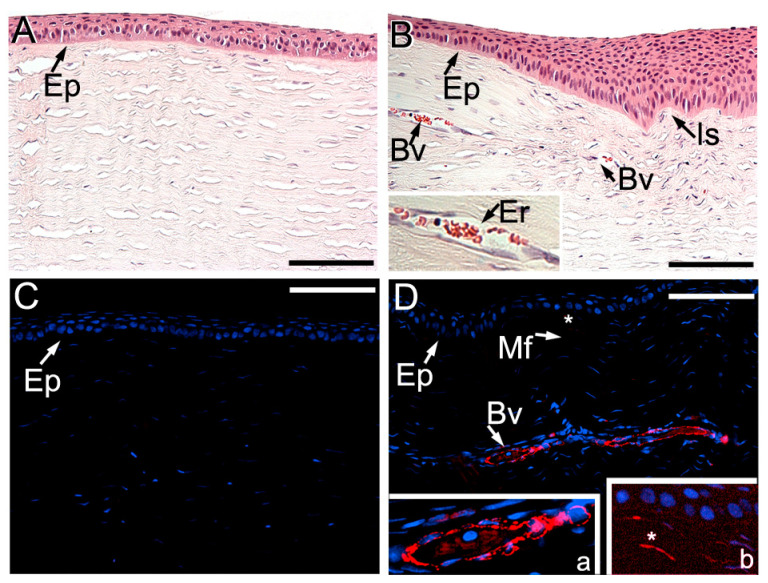
Representative images of the uninjured (**A**,**C**) and injured (**B**,**D**) cornea sites from the ACA group. Panels (**A**,**B**) show hematoxylin and eosin (H&E)-stained tissue samples. At the same time, (**C**,**D**) depict samples immunostained with the anti-αSMA antibodies (red) and nuclei (blue) stained with 4′,6-diamidino-2-phenylindole (DAPI). Epithelia (Ep) blood vessels (Bv), erythrocytes (Er), and the incision site (Is) are indicated. In panel (**D**), insert “a” shows a more detailed view of a blood vessel, and insert “b” depicts an αSMA-positive myofibroblast (Mf, asterisks). Bars = 50 μm. Note: the brightness in the entire insert “b” has been enhanced digitally to clearly outline the myofibroblasts.

In contrast to uninjured corneas (Figure 3A,C), the vascularization of injured stroma was also demonstrated in histological samples, including those immunostained with the antialpha smooth muscle actin (αSMA) antibodies (Figure 3B,D, insert “a”). Of note was the observation that, in addition to the blood vessels’ walls, there were sparse αSMA-positive myofibroblasts present in the injured stromata in all injury sites (Figure 3D, insert “b”). No similar cells were seen in the stromata of uninjured corneas (Figure 3C). We observed no signs of damage to the corneal epithelia eight weeks after injury in any of the analyzed groups.

### 2.3. Histopathology of Injury Sites

**Figure 4:**
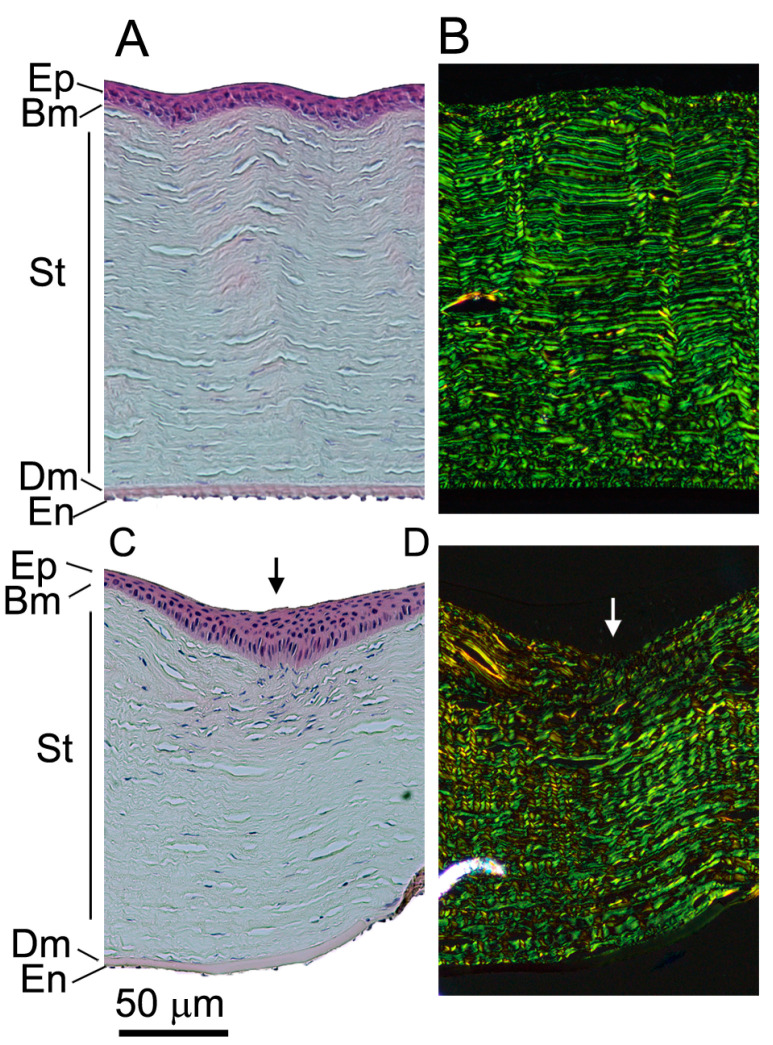
H&E (**A**,**C**) and collagen-specific picrosirius red (**B**,**D**) staining of the uninjured (**A**,**B**) and injured (**C**,**D**) sites. Please note that panels (**C**,**D**) are representative images for the injured sites from the ACA-treated and Ctr groups. Epithelium (Ep), Bowman’s layer (Bm), stroma (St), Descemet’s membrane (Dm), endothelium (En), and the incision site (arrows) are indicated.

Hematoxylin and eosin (H&E) staining demonstrated normal morphology of corneal layers in the Un group (Figure 4A). As expected, picrosirius-red-stained corneal stroma included uniform collagen fibrils (Figure 4B). In contrast, the injury sites, in ACA-treated and control groups, featured wedged epithelium (Figure 4C) and aberrant fibrils (Figure 4D). Moreover, we observed myxoid stroma and compact collagen fibrils in the injury sites of the ACA and Ctr groups.

**Figure 5:**
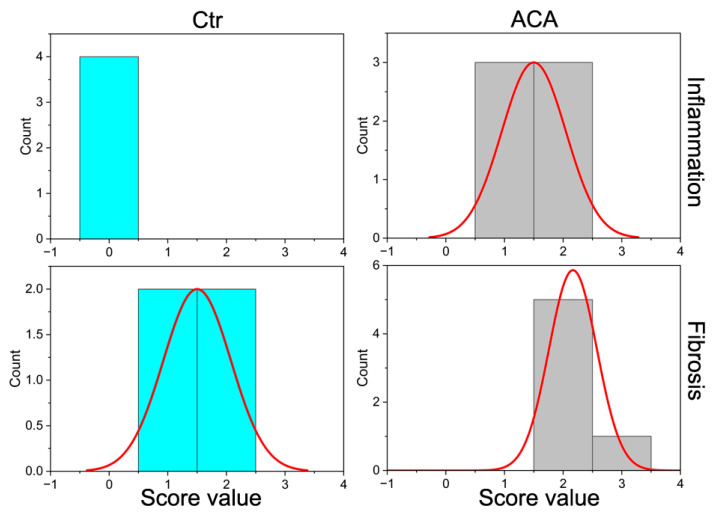
Histograms showing the distribution of scores for inflammation and fibrosis in the injury sites of the control (Ctr) and ACA-treated (ACA) groups.

We performed histopathological analysis of fibrosis extent and infiltration of the inflammatory cells into the injury sites. Table 2 shows no significant differences in distributions of the fibrosis scores for the Ctr (mean rank = 3.75) and ACA (mean rank = 6.67) groups. In contrast, distributions of the infiltration scores of inflammatory cells for these groups were significantly different (Ctr mean rank = 2.5 and ACA mean rank = 7.5). Figure 5 illustrates the distribution of fibrosis and inflammation scores. Neither fibrotic nor inflammatory changes were observed in uninjured corneas.

### 2.4. Collagen Fibrils’ Morphology and Organization

Utilizing the CF-FIRE and FibrilTool image analysis computer programs, we measured the crucial morphological parameters of the collagen fibrils in the scar (Sc), scar-adjoining (Ad), and uninjured (Un) cornea regions. A one-way ANOVA test was conducted to determine if essential characteristics of fibrils, including length, width, straightness, and anisotropy, were different among the Sc-ACA (*n* = 6), Sc-Ctr (*n* = 4), Ad-ACA (*n* = 6), Ad-Ctr (*n* = 4), and Un (*n* = 10) groups.

#### 2.4.1. Length

Microscopic analyses indicated that there was a statistically significant difference in the length of the fibrils among the analyzed groups (*F*(4,25) = 13.236, *p* < 0.0001). A post hoc Tukey’s test revealed that this difference was attributed to a significant reduction in the fibrils’ length in the Sc-ACA group vs. the Ad-ACA (*p* = 0.001) and Un (*p* < 0.0001) groups and the Sc-Ctr group vs. the Ad-ACA (*p* = 0.001) and Un (*p* < 0.001) groups.

#### 2.4.2. Width

There were no statistically significant differences in the widths of the fibrils among the analyzed groups (*F*(4,25) = 0.907, *p* = 0.475).

#### 2.4.3. Straightness

We also measured the straightness of the fibrils in the analyzed groups (the value of “1” represents straight fibrils). Data analysis indicated that there was a statistically significant difference in the straightness of the fibrils among the analyzed groups (*F*(4,25) = 10.904, *p* < 0.0001). A post hoc Tukey’s test revealed that this difference was attributed to a significant reduction in the fibrils’ straightness in the Sc-ACA group vs. the Un group (*p* < 0.001), the Sc-Ctr group vs. the Ad-ACA (*p* = 0.032) and Un (*p* < 0.001) groups, and the Ad-Ctr group vs. the Un group (*p* = 0.016).

**Figure 6:**
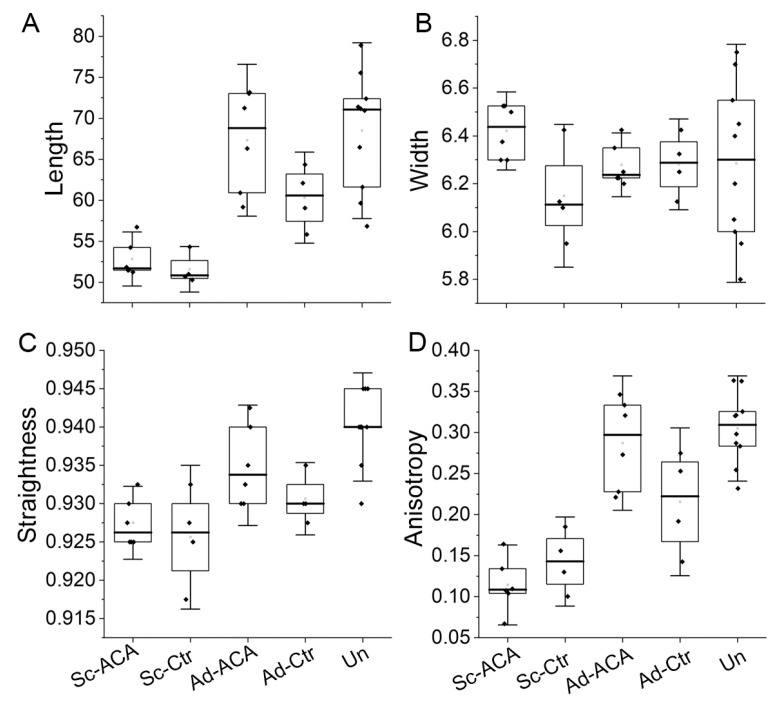
Box plots representing the length (**A**), width (**B**), straightness (**C**), and anisotropy (**D**) of the fibrils from the Sc-ACA, Sc-Ctr, Ad-ACA, Ad-Ctr, and Un sites. The interquartile range between the 25th and 75th percentiles determines each box. The lines within the boxes represent the medians, while the whiskers delineate the SD values.

**Figure 7:**
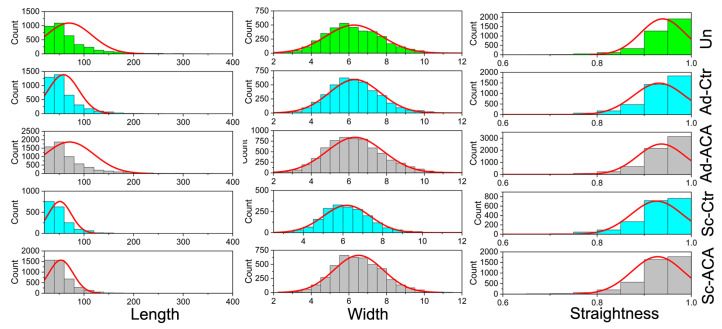
Histograms showing the distribution of the values of the collagen fibril length, width, and straightness measured in the Sc-ACA, Sc-Ctr, Ad-ACA, Ad-Ctr, and Un sites of the corneas.

Figure 6A–C summarizes the above results. In addition, we present histograms illustrating the distribution of the analyzed parameters’ values (Figure 7).

#### 2.4.4. Anisotropy of the Fibrils

In addition to the above parameters, the organization of collagen fibrils, expressed as a theoretical anisotropy score, was also evaluated by employing the ImageJ software, version 1.53t, that included the FibrilTool plug-in [25,26].

Unlike the CF-FIRE software, version 2.0 Beta, which measures the length, width, and straightness of identified individual fibrillar elements, the FibrilTool considers the entire fibril population present in a region of interest (ROI) and calculates their anisotropy score, where the value of “1” signifies fibrils arranged directionally. Lower anisotropy scores indicate varying degrees of fibril disorganization.

Data analysis indicated that there was a statistically significant difference in fibril anisotropy among the analyzed groups (*F*(4,25) = 22.773, *p* < 0.0001). A post hoc Tukey’s test revealed that this difference was attributed to a significant reduction in the anisotropy in the Sc-ACA group vs. the Ad-ACA (*p* < 0.00001), Ad-Ctr (*p* = 0.015), and Un (*p* < 0.00001) groups. Moreover, this difference was also attributed to a significant reduction in the anisotropy in the Sc-Ctr group vs. the Ad-ACA (*p* < 0.001) and Un (*p* < 0.0001) groups and the Ad-Ctr group vs. the Un group (*p* = 0.021).

Figure 6D summarizes the results of anisotropy assays.

### 2.5. Fourier Transform Infrared Spectroscopy (FTIR)-Based Collagen and PG Content Assays

#### 2.5.1. Relative Collagen Content

**Figure 8:**
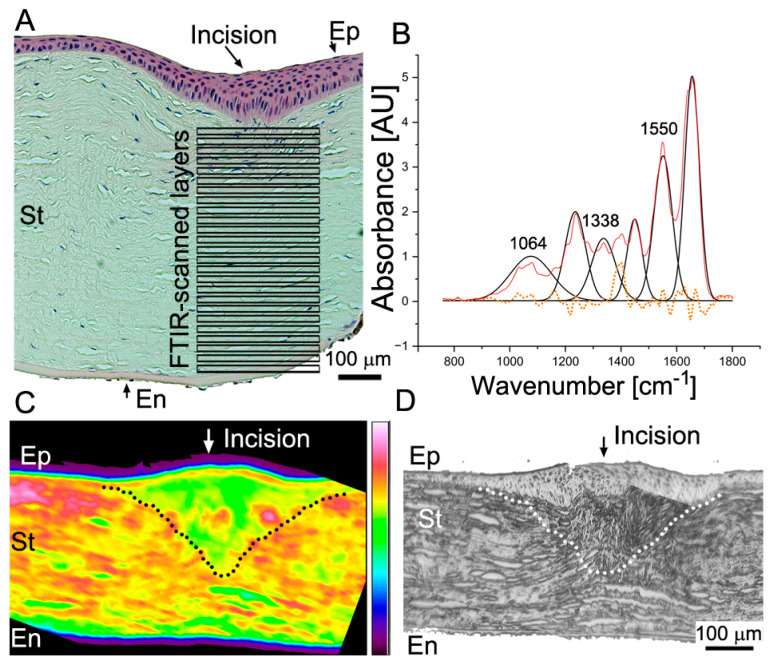
FTIR spectroscopy assays of the corneal stromata. (**A**) An H&E-stained cornea depicting the scar neotissue area in the incision site. Indicated boxes delineate individual layers utilized to obtain 2D correlation. (**B**) A representative FTIR spectrum collected from one of the layers depicted in (**A**). The peaks used in this study to quantify collagen and PGs are indicated. The red line shows the FTIR spectrum data collected from the stroma layer; the black line represents a curve fitted to the raw spectra; and symmetrically distributed residuals (dotted line) indicate a proper curve fit. (**C**) An FTIR spectroscopy map shows an average absorbance at the area depicted in (**A**). (**D**) A visible light image of the area shown in (**C**). The delineated areas in (**C**,**D**) highlight the scar neotissue formed in the incision site. Symbols: Ep, epithelium; St, stroma; En, endothelium; AU, arbitrary units.

Utilizing FTIR spectroscopy, we measured the relative content of collagen in the injured and injury-adjoining sites (Figure 8A,C,D). Collected spectra were analyzed using curve fitting (Figure 8B).

A one-way ANOVA was conducted to determine if the collagen content was different for the Sc-ACA (*n* = 6), Sc-Ctr (*n* = 4), Ad-ACA (*n* = 6), Ad-Ctr (*n* = 4), and Un (*n* = 10) groups.

**Figure 9:**
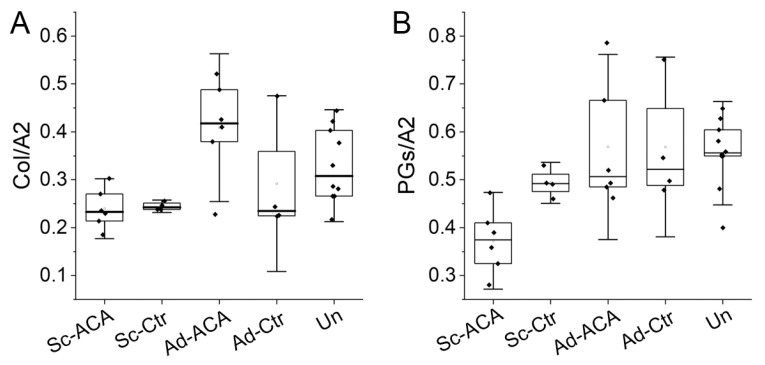
Box plots representing the relative collagen content (**A**) and the relative PG content (**B**) in the Sc-ACA, Sc-Ctr, Ad-ACA, Ad-Ctr, and Un sites. The relative collagen content was calculated as the ratio of the FTIR-based collagen-derived peak (Col, centered around 1338 cm^−1^) area and the amide II (A2)-derived peak (centered around 1550 cm^−1^) area. The relative PG content was calculated as the ratio of the FTIR-based PG-derived peak (centered around 1064 cm^−1^) area and amide II-derived peak area. The interquartile range between the 25th and 75th percentiles determines each box. The lines within the boxes represent the medians, while the whiskers delineate the SD values.

These analyses indicated a statistically significant difference in the relative collagen content among the analyzed groups (*F*(4,25) = 4.279, *p* = 0.009). A post hoc Tukey’s test revealed that this difference was attributed to a significant reduction in the relative collagen content in the Sc-ACA group vs. the Ad-ACA (*p* = 0.001) and Un (*p* = 0.04) groups; Sc-Ctr vs. the Ad-ACA group (*p* = 0.004); and Ad-Ctr group vs. Ad-ACA group (*p* = 0.03) (Figure 9A).

#### 2.5.2. Relative PG Content

FTIR spectroscopy was also employed to determine the relative PG content. One-way ANOVA results indicated a statistically significant difference in the relative PG content among the analyzed groups (*F*(4,25) = 5.208, *p* = 0.003). A post hoc Tukey’s test revealed that this difference was attributed to a significant reduction in the relative PG content in the Sc-ACA group vs. the Sc-Ctr (*p* = 0.048), Ad-ACA (*p* = 0.001), Ad-Ctr (*p* = 0.002), and Un (*p* = 0.001) groups (Figure 9B).

### 2.6. Two-Dimensional Correlation FTIR Spectroscopy (2DCS)

**Figure 10:**
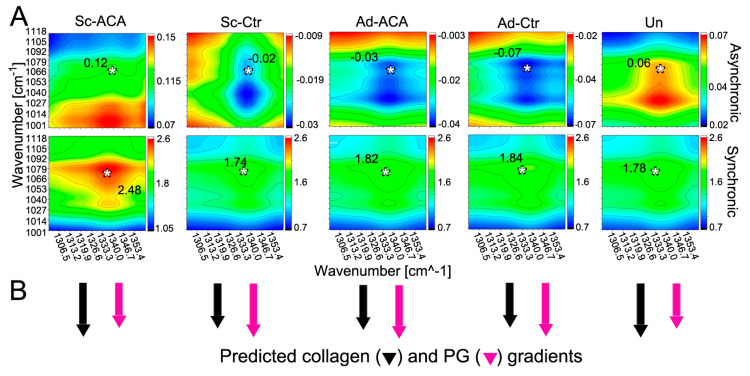
(**A**) Two-dimensional correlation FTIR spectroscopy (2DCS) asynchronous and synchronous maps showing the correlation values of the collagen and PG changes occurring along the stroma depth in the Sc-ACA, Sc-Ctr, Ad-ACA, Ad-Ctr, and Un cornea regions. Please note that crucial 2D correlation values at ν1/ν2 coordinates are indicated in each panel. These values are at the collagen-derived (ν1; centered around 1338 cm^−1^) and PG-derived (ν2; centered around 1064 cm^−1^) cross-peaks indicated by asterisks (*). (**B**) A graphic presentation of the stroma-depth-dependent collagen and PG changes. The arrows show whether stroma-depth-dependent changes in collagen and PG signals occur in the same or opposite directions. A longer arrow indicates that the changes it represents (e.g., in collagen) occur before those of an analyzed partner (e.g., in PGs).

We also analyzed the synchronous and asynchronous 2D correlations between changes occurring in collagen and PGs as a function of the corneal stroma depth (Figure 8A). These changes were analyzed in the Sc, Ad, and Un regions of the ACA and Ctr groups. Figure 10 demonstrates the correlation values for the collagen and PG cross-peaks (centered around 1338/1064 cm^−1^ X/Y axes coordinates) obtained from the 2D correlation maps (Figure 10).

Based on the synchronous correlation assay, in the Un cornea region, the depth-dependent changes in the collagen-specific and PG-specific FTIR-derived signals co-occurred in the same direction. In addition, the results of the asynchronous 2D correlation indicated that in the Un group, changes in the collagen signals slightly preceded those in the PG signals. Similar results were obtained for the Sc-ACA group.

In contrast, synchronous and asynchronous correlation assays of the Sc-Ctr, Ad-ACA, and Ad-Ctr groups suggested that the depth-dependent changes in the collagen content occurred after those observed for the PGs.

Please note that although we considered the 1064 cm^−1^ peak a PG-specific indicator, the spectral characteristics of carbohydrates encompass a broader region between the wavenumbers 984 and 1140 cm^−1^.

## 3. Discussion

### 3.1. Corneal Scarring

The present study focuses on scar neotissue formed after eight weeks in the incision wounds created in rabbit corneas. During this study, we also analyzed the effects of antifibrotic ACA on collagen-rich corneal scar tissue formation [20,22,27]. Utilizing microscopic and spectroscopic methods, we measured two crucial elements of the corneal stroma: collagen and PGs.

A canonical understanding of collagen-rich tissue healing, including the cornea, is that inflammation drives this process [28]. As inflammatory cells migrate to the injury sites, they secrete various factors, most notably transforming growth factor β1 (TGFβ1), promoting collagen-rich scar tissue formation and differentiation of the stromal keratocytes into myofibroblasts that accelerate scar tissue formation [29].

While the scar maintains tissue integrity, its excessive growth causes many medical problems. Excessive corneal scars, mainly when formed in the central areas, may obscure vision and, in extreme cases, even cause blindness. Disorganized alignment of newly formed collagen fibrils and neovascularization of scar neotissue is the main culprit causing transparency loss [30,31].

Clinically applied methods to reduce corneal scarring include topical anti-inflammatory steroids, which affect numerous processes associated with scar formation [32]. Moreover, antiproliferative mitomycin C and 5-fluorouracil are also used to minimize corneal scarring [13].

Because the outcomes of these pharmacological treatments are not always effective and may be associated with significant risks, surgical procedures may be performed to reduce or eradicate the opaque scar, including keratectomy, phototherapeutic keratectomy, lamellar keratoplasty techniques, and others. These procedures, however, carry many risks, including recurrence of scarring, infection, graft rejection or failure, corneal thinning, astigmatism, and many others [33,34].

Consequently, scientists have developed and tested novel experimental methods to reduce corneal scarring [13]. For instance, gene therapy techniques with viral-vector-based gene delivery were proposed to target profibrotic TGFβ1-dependent pathways [11,13]. Moreover, researchers have considered repurposing some drugs approved for other disorders and applying them to limit corneal scarring. Examples include losartan (designed to treat high blood pressure), fingolimod (used to treat certain forms of multiple sclerosis), and pirfenidone (developed to treat idiopathic pulmonary fibrosis) [13].

### 3.2. Antiscarring ACA to Treat Corneal Scarring

Our group has demonstrated that targeting collagen fibrillogenesis with ACA attenuates scar formation and does not cause any side effects in animal-based preclinical models of fibrotic scarring [22,27]. These earlier studies targeted posttraumatic scarring in tissues with a relatively low degree of collagen fibril organization. In contrast, here, we were interested in the utility of ACA in limiting excessive scarring in the cornea, whose light-transmitting functions depend on a highly organized collagenous matrix. In particular, we tried to determine if ACA could improve collagenous architecture in neotissue formed in response to cornea injury.

Consequently, using a rabbit model, we studied the impact this antibody has on corneal scar formation, composition, and organization of its crucial elements, i.e., collagen fibrils and PGs. We defined relevant and measurable parameters to describe the scar neotissue formed in the injury sites in the presence of ACA. We also analyzed the impact of this antibody on areas adjoining the incision margins.

#### 3.2.1. In-Life ACA Effects

In-life analyses of the depth, opacity, edema, and vascularization indicated that eight weeks after injury, only the vascularization scores for the wounded areas in the ACA-treated corneas were significantly higher than in PBS-treated control. Subsequent histopathological assays confirmed this trend. However, while in-life observations demonstrated the presence of vasculature in the wounded corneas of the control group, subsequent histopathological analyses did not, perhaps due to the random sampling of injured tissue used for histology.

We cannot explain the difference in the extent of scar vascularization between the ACA-treated and control groups, except that this difference could only result from injecting the protein-rich ACA solution in the treated group vs. the PBS solvent injected into the control. Please note we decided to use PBS as a control according to recommendations on testing monoclonal antibodies, which advise applying a solvent only instead of a solution of a non-reactive antibody [35,36]

It is likely, however, that the presence of the protein-rich ACA solution caused a more significant inflammatory response than PBS, thereby promoting neovascularization. A substantial increase in the inflammation score (Table 2, Figure 5) for the ACA-treated group observed with histopathological assays supports this notion. Still, even with a higher inflammation score, the fibrosis scores in the ACA and Ctr groups did not differ significantly (Table 2, Figure 5).

Although our research represents early steps in testing novel approaches to limiting corneal scarring, we cannot exclude the possibility that using antibody-based therapies to reduce corneal scar formation could cause unwanted side effects. Hence, further studies are warranted to clarify this problem.

#### 3.2.2. ACA Effects on ECM Formed at Injury Sites

Application of ACA, however, demonstrated some potentially positive effects of this antibody on reducing corneal scar deposits. For instance, utilizing FTIR spectroscopy, we determined that the relative amounts of collagen in the neotissue formed in the incision sites of the ACA-treated and control groups were similar. However, compared to the Ad and Un sites, these amounts trended toward lower values. Of interest was the observation that in the Sc-ACA, but not the Sc-Ctr, group, the relative collagen content was significantly smaller than in the corresponding Un sites. This result may indicate that ACA treatment reduces the relative collagen content due to attenuation of fibrillogenesis and consequent degradation of ACA-blocked collagen molecules not incorporated into fibrils. Our earlier studies support this notion, demonstrating accelerated degradation of free, i.e., not fibril-associated, collagen molecules [22].

Although ACA specifically targets the assembly of collagen fibrils, we also analyzed its impact on the relative amount of PGs. The rationale for these assays is that collagen fibrils form a matrix template needed to coassemble and retain other components, including PGs, within the extracellular matrix (ECM). The collagen–PG coassembly is particularly important in developing a transparent corneal architecture [37,38,39]. Our FTIR-spectroscopy-based results demonstrated a significant reduction in the relative amount of PGs in the corneas’ Sc regions of the ACA-treated group.

These data suggest that ACA-based reduction of collagen fibrils could be a reason for the observed decrease in the scar neotissue’s PGs in the ACA-treated group. Consequently, we propose that the ACA not only reduces the collagenous scar components but could also have much broader antiscarring effects.

#### 3.2.3. ACA Impact on the Fibrillar Architecture of Scar Neotissue

While the orthogonal arrangement of layers formed by parallel collagen fibrils interlacing with PGs facilitates corneal transparency, the disturbed fibrillar architecture of corneal scars alters it [11]. Consequently, we studied crucial fibril morphology and organization parameters in the scar neotissue and adjoining regions to determine the effects of ACA-mediated attenuation of neofibrillogenesis during corneal wound healing.

A remarkable observation was that the fibrils’ length decreased significantly compared to uninjured control in the scar neotissue formed in the incision sites created in the ACA and Ctr groups. Moreover, an unexpected observation was that in the Ctr group, the length of the fibrils in the regions adjoining the injury site was significantly shorter than in the Un sites. This observation may suggest that the effects of incision injuries in the cornea may “spill over” into neighboring uninjured regions beyond the incisions’ margins.

Remarkably, the length of the fibrils formed in the Ad regions of the ACA-treated group did not change and was similar to that of the Un group. We cannot exclude the possibility that ACA-mediated attenuation of neofibrillogenesis prevented the formation of atypical new fibrils within the Ad regions [20].

Compared to the fibril straightness in the Ad-ACA group, which was similar to the straightness measured for the Un group, the fibril straightness values were significantly smaller in the Ad-Ctr regions. This observation further suggests that the presence of ACA attenuated the formation of atypical neofibrils via the mechanisms we described [20,22].

Unlike the fibrils’ length and straightness, their width did not change in any of the analyzed groups. Because diffuse light scattering obscures the width of fibrillar structures observed in a light microscope, transmission electron microscopy (TEM) of fibrils’ cross-sections is warranted to evaluate our light microscopy results.

Another group of parameters we analyzed defined the organization of collagen fibrils crucial for cornea transparency. Compared to the Un group, we demonstrated that the anisotropy score, which describes the fibril organization, decreased significantly in scar neotissue formed in the ACA and Ctr incision sites. This result indicates that fibrils that build the corneal scars lack the natural organization of lamellae.

Moreover, we also observed a significant decrease in the anisotropy score in the Ad regions of the Ctr group. This result and changes in fibril morphology described above further support our conclusion that aberrations of the corneal fibrillar architecture go beyond the incisional margins.

Because, unlike in the Ctr group, the anisotropy of the fibrils present in the Ad regions of the ACA group was similar to that of the Un group, we further propose that ACA attenuated aberrations of the collagenous matrix caused by the incisional wounds in the rabbit cornea.

Overall, our observations of the structure and organization changes of the collagen fibrils formed in untreated injury sites support those reported by other authors. For instance, Dawson et al. observed similar changes in the morphology and organization of collagen fibrils formed in corneal wounds caused during photoreactive keratectomy procedures [40]. The authors used electron microscopy to observe sparse collagen fibrils without defined organization. Moreover, they observed alterations in PG organization, including lack of binding with collagen fibrils and size changes.

#### 3.2.4. Spectroscopic Assays of Scar Neotissue Formed in ACA’s Presence

To extend our understanding of postinjury alterations of the corneal stroma structure, we applied 2DCS to determine potential changes in the spatial distribution of collagen and PGs. The spatial distribution of these elements dictates crucial mechanical properties of the cornea, maintains proper hydration, and facilitates the transparency of this tissue [6].

Although scientists agree on peculiar collagen and PG distribution gradients along the anterior–posterior stroma axis, their detailed characteristics remain obscure. For instance, Castoro et al. studied water gradients across the bovine cornea in the context of PG distribution [41]. They observed a negative chondroitin-4-sulfate gradient and a positive keratan sulfate gradient along the anterior–posterior stroma axis. Similarly, Puri et al. indicated a positive gradient of lumican, decorin, and biglycan distribution [42].

Studies have demonstrated that the quality, quantity, and distribution of PGs change during the healing of wounded corneas, altering the average distribution gradients [43,44]. However, the harmful effects of modified collagen and PG distribution are not unique to corneal healing. For instance, they have also been described for Scheie’s syndrome, a disorder characterized by corneal opacification due to abnormal systemic accumulation of glycosaminoglycans [45]. Moreover, PG alterations are associated with corneal ectasia, cornea plana, macular corneal dystrophy, and Marfan-syndrome-related ocular aberrations [42].

Similarly, studies have demonstrated depth-dependent changes in the collagenous architecture of the cornea. For instance, microscopic assays of the cornea’s central region revealed the fan-like distribution of consecutive rotated fibrous lamellae in the anterior stroma, with a fixed superior–inferior orthogonal gridwork in the deeper layers of the tissue [46].

Here, we used 2DCS to measure the correlation of quantitative changes in the collagen and PGs occurring along the anterior–posterior stroma axis. Compared to the microscopic methods that usually require ECM element fixation and staining, FTIR spectroscopy analyzes the chemical composition signatures of unprocessed ECM; thus, we obtained more biologically relevant information on their codistribution in the context of the corneal depth.

Although microscopic studies have suggested various positive or negative anterior–posterior gradients of different PG species, as indicated above, our synchronous correlation data suggest that in uninjured corneas, the net collagen and PG contents change in the same direction (Figure 10B). Because the signs of synchronous and asynchronous correlation values from the Un group were the same, we concluded that the depth-dependent collagen (ν1) quantitative changes occurred before those observed for PGs (ν2).

Similar collagen and PG gradient patterns were observed in scar neotissue treated with ACA (Sc-ACA). In contrast, in the Ad-ACA, Sc-Ctr, and Ad-Ctr regions, the depth-dependent collagen changes occurred after those measured for PGs.

The 2DCS results seem to refine our microscopic observations that corneal injuries disturb the fine architecture of the stromal matrix in the regions adjoining the incisional wounds. Moreover, they indicate that scar neotissue formed in the corneal injury sites lacks proper gradients of interlacing collagen and PG elements. Consequently, we suggest that depth-dependent collagen and PG distribution gradients are crucial corneal features that contribute to the transparency of this tissue.

Our results indicate that ACA preserves the collagen and PG distribution patterns in the neotissue formed in injury sites. Despite this preservation, the scars’ opacity in the Sc-ACA sites was relatively high. This observation may suggest that maintaining native-like collagen and PG distribution gradients cannot compensate for poor alignment of the fibrils formed within the scar neotissue.

#### 3.2.5. Limitations and Clinical Implications

We have identified a few limitations of our study: (i) The results of this study are only relevant to nonpenetrating incisional corneal wounds. (ii) The postoperative healing period of eight weeks was relatively short; longer healing times are warranted to fully comprehend the long-term ACA effects. (iii) The study would benefit from assays of mechanical properties of scar neotissue to determine ACA’s impact on tissue integrity. (iv) Our quantitative assays of collagen and PGs provide the relative amounts of these molecules; in future studies, biochemical assays of neotissue components will provide the actual amount values per dry mass unit of the cornea. (v) Finally, the ACA we employed in the rabbit model is the humanized IgG4 variant. Thus, we cannot exclude the possibility that the antibody’s xenogeneic character was partly responsible for inflammatory responses and increased neovascularization.

The findings from this study have several clinical implications. First, in nonpenetrating injury to the cornea, ACA may modify the consequent fibrosis that results in corneal opacity and decreased vision for patients. Further research would help determine the optimal timing, regimen, and mode of delivery of ACA to optimize healing and reduce visual morbidity. Second, ACA may play a role in treating patients who develop corneal haze or fibrosis after surgical intervention, for example, after corneal collagen cross-linking or excimer laser treatment. These therapies are often associated with anterior corneal stromal haze, and ACA may modulate the development of this haze.

Considering that applying 5-fluorouracil and mitomycin-C is a standard practice to reduce corneal scarring, it will be essential to compare the effectiveness of these drugs with that of ACA. However, based on our recent study on using ACA to limit scar formation associated with glaucoma surgery, we already know that compared to mitomycin C, ACA offers some advantages. For instance, compared to mitomycin-C-treated injury, we observed a significantly lower number of profibrotic cross-links in the ACA-treated areas [27]. Further studies comparing ACA with 5-fluorouracil and mitomycin-C will show the advantages and disadvantages of different modes of reducing fibrosis in corneal scarring.

Despite the need for additional research, our pilot study contributes significantly to understanding structural changes of the corneal stroma due to injury and subsequent healing. In addition, it sheds new light on the antiscarring utility of directly targeting corneal fibrillogenesis with rationally engineered ACA.

## 4. Materials and Methods

### 4.1. ACA Preparation

Recombinant humanized ACA was produced in Chinese hamster ovary (CHO) cells grown in suspension in a bioreactor [23]. ACA was purified by affinity chromatography using protein-L resin. Following purification, ACA was concentrated and then sterilized by filtration [20,22]. ACA stock was prepared at 30 mg/mL in phosphate-buffered saline (PBS).

### 4.2. Surgical Procedure and ACA Application

A total of 10 New Zealand White rabbits (5 males and 5 females) between 8 and 12 months old were employed for this study. The rabbits’ eyes were divided into the following groups: (i) injured and treated with ACA (ACA group, number of eyes *n* = 6); (ii) injured and treated with PBS only (Ctr group, number of eyes *n* = 4); and (iii) uninjured and nontreated (Un group, number of eyes *n* = 10).

As no data on the collagen-reducing ACA’s effects exist for the cornea, we estimated the number of animals needed for our study based on results obtained from our studies of arthrofibrosis [23]. We performed power analysis using GraphPad StatMate version 2.00 for Windows (GraphPad Software, San Diego, CA, USA) [23]. In brief, we demonstrated that ACA decreased the collagen content per dry tissue mass unit from 79% (untreated injured control joint) to 59% (ACA-treated injured joints). Considering a standard deviation of 8% and a difference of 20%, we concluded that a sample size of 5 in each group would have 95% power to detect a difference between means of 21.5% with a significance level (alpha) of 0.05 (two-tailed).

The Institutional Animal Care and Use Committee of Thomas Jefferson University reviewed and approved the study protocol TJU IACUC protocol #21-08-420, approved on 13 December 2021). All animals were treated under the Association of Research in Vision and Ophthalmology (ARVO) Statement for the Use of Animals in Ophthalmic and Vision Research.

The anesthetized rabbits were placed under a surgical microscope. A drop of 5% betadine and topical lidocaine 1–2% gel was placed in the operated left eye. A sterile speculum was placed to open the eye. A diamond blade set at 100 microns created a linear horizontal corneal incision approximately 2 mm from the superior limbus, extending about 75% of the limbus-to-limbus length at this location. The incision was made paracentral to avoid affecting visual acuity (Figure 1). The right eye served as the uninjured control.

Immediately after the incision, 300 μg of ACA in a 100 μL volume was injected intrastromally into the incision’s margins. ACA was injected on both sides of the linear incision, and attempts were made to distribute the medication evenly along the incision length. The Ctr group received 100 μL of PBS. Subsequently, a drop of triple antibiotic (neomycin, polymyxin B, and bacitracin) was placed in the injured eyes.

A second round of antibody injections was performed after one week; the same ACA concentration and injection procedures were applied.

### 4.3. Postoperative Monitoring of the Corneas

The anterior examination was performed at postoperative weeks one, two, four, six, and eight under anesthesia using a slit lamp. An experienced clinical ophthalmologist examined masked treatment groups and graded the scar depth, opacity, edema, and vascularization. The grading system parameters are presented in Table 1.

Moreover, we measured the surface area of any epithelial defect, defined as the uptake of fluorescein stain in the area of injury, and expressed it in millimeters.

The results of these measurements carried out in week eight were analyzed using the nonparametric Mann–Whitney U test (IBM SPSS Statistic for Windows, version 26, IBM Corp., Armonk, NY, USA). In all assays, statistical significance was defined as *p* ≤ 0.05. Results were tabularized and presented in the form of histograms.

### 4.4. Tissue Collection

Following euthanasia, the rabbits’ eyes were enucleated and submerged in 4% paraformaldehyde. Each eye was examined under the dissecting microscope. Then, the anterior segment of the eye was removed by opening the eye equatorially. A central cross-section was taken in the plane to incorporate the incision site perpendicular to the orientation of the incision.

Following routine tissue processing and paraffin embedding, each block was sectioned in three-step levels at 50 µm intervals. Five 5 µm thick sections were obtained at each level and stained with H&E, Masson’s trichrome, or periodic acid-Schiff dyes. Additional unstained preparations were used to stain collagen fibrils with picrosirius red [27].

### 4.5. Histopathology of the Eyes

H&E-stained sections were analyzed blind by an ocular pathologist (T.M.), who scored the anterior segment for the presence of inflammatory cells, vascularization, and the extent of fibrosis in the incision area [27]. In addition to H&E staining, the presence of blood vessels was confirmed with immunostaining using anti-αSMA antibodies, as we have described elsewhere [47].

### 4.6. Analysis of Collagen Fibrils’ Morphology and Organization

We selected two areas of interest per each injured cornea: (i) a region of scar neotissue, referred to as Sc, present within the incision site and (ii) the incision-adjoining region, referred to as Ad, located, on average, 2 mm from the incision site. Moreover, we analyzed the corneas from uninjured (Un) eyes. For each area, we analyzed multiple ROIs.

Because the corneal stroma and scar tissue predominantly comprise collagen fibrils, the histological sections were stained with collagen-specific picrosirius red dye [27,48,49]. Then, employing a polarizing microscope (Eclipse LV100POL, Nikon Inc., Melville, NY, USA) and the NIS Elements software version 3.22.14 (Nikon Inc.), we captured digital images of collagen fibrils present in selected ROIs of the Sc, Ad, and Un regions. Subsequently, we measured collagen fiber length and width (expressed in pixels) for each ROI using the segmentation software CT-FIRE version 2.0 Beta (Laboratory for Optical and Computation Instrumentation, University of Wisconsin, Madison, WI, USA) [50,51,52]. The same software was used to measure the straightness of the fibrils, where a value of “1” represented perfectly straight fibrils and the smaller values represented varying degrees of the fibrils’ curvature.

At the end of the analysis of the Sc, Ad, and Un areas, the software returned the ROI means for each parameter, i.e., the length, width, and straightness. Subsequently, the ROI means were averaged for each rabbit. Next, we applied one-way ANOVA to determine the statistical significance of the differences between analyzed parameters in the ACA-treated and Ctr groups (IBM SPSS Statistic for Windows, version 26, IBM Corp., Armonk, NY). The results were described and presented graphically in the form of box plots. In addition, data from all individual fibrils were presented as histograms (OriginPro version 2023, OriginLab Corporation, Northampton, MA, USA).

The spatial organization of collagen fibrils, expressed as a theoretical anisotropy score, was evaluated using the ImageJ software that included the FibrilTool plug-in [25,26]. The score of “1” represents perfectly anisotropic, i.e., ordered parallel fibers, while smaller values indicate various degrees of random order [25]. Data were analyzed and presented as described above. Because the FibrilTool returns a single anisotropy score representing the global organization of the fibrils within the analyzed ROIs, no histograms for the anisotropy scores were contemplated.

### 4.7. Fourier Transform Infrared Spectroscopy (FTIR)-Based Collagen and PG Content Assays

In biological studies, FTIR spectroscopy utilizes infrared light to analyze the chemical composition of tissues [53]. A significant advantage of FTIR spectroscopy of tissue samples over histological and biochemical methods is that it is nondestructive and performed on intact, label-free specimens. Thus, as we demonstrated earlier, FTIR spectroscopy provides quantitative and qualitative information about measured elements in the context of their spatial arrangement within analyzed tissues, including the eyes [27,54].

The central principle of FTIR spectroscopy is the generation of spectral data derived from vibrations of molecular bonds subjected to infrared beams. Because tissue macromolecules, including collagens and PGs, have unique spectral signatures, it is possible to quantify them in the spatial context of tissue architecture [55,56].

We used this method to measure the relative collagen and PG content in the corneas’ Sc, Ad, and Un areas. Based on the FTIR spectra, we determined the areas of the collagen-specific peaks (arising due to CH_2_ wagging vibrations of proline side chains) centered around a 1338 cm^−1^ wavenumber and the carbohydrate region (984–1140 cm^−1^) that included sulfated PG-associated peaks (arising from the symmetric stretching of the -SO_3_ groups) centered around a 1064 cm^−1^ wavenumber. We also utilized an internal reference of a protein-derived amide II peak (from the N-H bending vibration) centered around a 1550 cm^−1^ wavenumber. Consequently, the collagen/amide II and PGs/amide II ratios were measured to determine the relative content of these crucial corneal components [22,57,58,59].

For the FTIR spectroscopy, paraffin-embedded 5 μm thick tissue sections were placed on the MirrIR low-e microscope slides (Kevley Technologies, Chesterland, OH, USA). Then, an FTIR spectrometer (Spotlight 400, Perkin Elmer, Waltman, MA, USA) was used to analyze the ROIs in the corneas, localized in the Sc, Ad, and Un areas. The measurements were conducted in the 4000 to 748 cm^−1^ wavenumber spectral range at a pixel resolution of 25 μm, 8 scans per pixel, and a spectral resolution of 4 cm^−1^. The Spectrum Image software, version R1.8.2.0413, created coadded spectra from scanned ROIs (PerkinElmer, Inc., Waltman, MA, USA).

Overlapping FTIR spectra peaks were deconvoluted and analyzed based on the second-order derivative spectra and predetermined bell-type Gaussian peak fitting function using the OriginPro software (OriginPro version 2023) [22,60,61].

One-way ANOVA was conducted to determine the significance of the difference in the relative collagen and PG contents among the analyzed groups (IBM SPSS Statistic for Windows, version 26). In all assays, statistical significance was defined as *p* ≤ 0.05. Results on the relative collagen and PG contents were described and presented in the form of box plots (OriginPro, version 2023).

### 4.8. Two-Dimensional Correlation FTIR Spectroscopy (2DCS)

In addition to quantifying the corneal stroma’s overall collagen and PG contents, we utilized 2DCS. In brief, 2DCS is a technique used to extract useful information from a set of spectral data obtained from a specimen exposed to an external perturbation. For example, the perturbation can modulate a solvent’s temperature, pH, or ionic strength, and a sample can be exposed to a gradually increasing temperature. During this process, a set of FTIR spectra is recorded. Because individual components of the analyzed sample, e.g., components A and B, may change their properties differently with changing temperature, collected spectra reflect these changes. Consequently, variations of spectral intensities in components A and B induced by, for example, temperature-dependent perturbations, are used to create 2D correlation spectra using cross-correlation analysis. The 2DCS method simplifies complex spectra consisting of many overlapping peaks, improves spectral resolution, and defines the sequence of events represented by the variations of spectral intensities [62,63].

Here, we collected FTIR spectra from consecutive layers of the corneal stromata in the Sc, Ad, and Un regions of the ACA-treated and control groups. The order of the layers was from the anterior toward the posterior stroma. We analyzed 25 consecutive layers for each area and collected 25 corresponding spectra.

In the context of the 2DCS rules, the “perturbance” we introduced was the change in the stroma depth. Because collagen and PGs are crucial components of the corneal stroma, we measured the depth-dependent correlation of the content of these molecules. Moreover, as the results of the 2DCS assays of consecutive stromal layers reflected the structural relationship between spatial collagen and PG arrangement, performing these assays allowed us to study potential changes in this relationship as an ACA treatment function. These measurements were based on averaged normalized spectral data from the corresponding corneal regions and layers. Two-dimensional maps showing the synchronous and asynchronous correlation of the collagen-specific and PG-specific peaks were generated using the OriginPro software. Based on these analyses, we obtained the 2D correlation values from the collagen-derived (1338 cm^−1^) and PG-derived (1064 cm^−1^) cross-peaks.

Please note that in synchronous 2D correlation assays, under the pressure of a perturbing factor (here, the cornea depth), positive correlation cross-peaks indicate a simultaneous change of the analyzed components in the same direction. In contrast, negative synchronous 2D correlation cross-peaks suggest that while one spectral intensity increases, the other decreases simultaneously [64].

In asynchronous 2D correlation, large correlation values indicate that the spectral intensities of the analyzed elements (here, collagen and PGs) vary independently in the context of perturbation (here, the corneal stroma depth). These high values indicate that corresponding spectral signals originate from separate moieties that respond differently to a common perturbation.

Unlike synchronous correlation, which represents simultaneous changes, asynchronous correlation reflects sequential changes in the signal intensities. In particular, asynchronous cross-peaks overlap if two dynamic spectral intensities vary, i.e., they are out of phase. Consequently, if the signs of both synchronous and asynchronous peaks present in the exact coordinates (here, 1338/1064 cm^−1^) are the same, the intensity change at wavenumber ν1 (here, the collagen-specific peak) occurs before that of wavenumber ν2 (here, the PG-specific peak). In contrast, when the signs of cross-peaks from synchronous and asynchronous correlations are different, the intensity variation of ν1 takes place after ν2 [64].

## Figures and Tables

**Table 1:** A grading system used to score the scar morphology parameters [24].

Scar Parameter	Grading Scores				
	1	2	3	4	5
Depth	Absent	Superficial ¼ of the cornea	Superficial ½ of the cornea	Superficial ¾ of the cornea	Full thickness
Opacity	Absent	Faint haze	Mild opacification	Moderate opacification	Severe opacification
Edema	Absent	Faint	Mild	Moderate	Severe
Vascularization	Absent	Vessels approaching but not reaching the incision edge	Vessels reaching but not crossing the incision edge	Vessels crossing the incision edge but with a minimal extension on the distal side	Vessels extending on both sides of the incision

**Table 2:** A summary of the results for corneal depth, opacity, edema, vascularization, fibrosis, and inflammation measurements carried out in injured corneas of the ACA and Ctr groups.

Mann–Whitney U Test Parameters	Injury Site Parameter					
	Depth ^a^	Opacity ^a^	Edema ^a^	Vascularization ^a^	Fibrosis ^b^	Inflammation ^b^
Mann–Whitney U	6.0	4.0	12.0	1.0	5.0	0.0
Z	−1.389	−1.826	0.0	−2.397	−1.845	−2.711
*p*	0.165	0.068	1.0	0.017	0.065	0.007
Mean ranks: Ctr; ACA	4.00; 6.50	3.50; 6.83	5.50; 5.50	2.75; 7.33	3.75; 6.67	2.50; 7.50

^a^ In-life observations; ^b^ histopathological observations. Z: The Z-value in the Mann–Whitney test is a test statistic that measures the difference between two independent samples. It is calculated as the ratio of the rank sum difference between two samples and the square root of the product of the sample sizes divided by two. The U-value represents the number of times observations in one sample precede observations in the other in the ranking.

## Data Availability

The data presented in this study are available on request from the corresponding author.
